# Evaluating the Need for Rotation Adjustments in Psychiatry Residency Programs: A Cross-Sectional Study

**DOI:** 10.1192/j.eurpsy.2025.2376

**Published:** 2025-08-26

**Authors:** J. P. Carrasco, J.-I. Etxeandia-Pradera, J. Esteve, C. Conde-Pumpido, B. Herraiz, E. Aguilar

**Affiliations:** 1 Hospital Provincial Castellón, Castellón; 2 Hospital Clínico Universitario de Valencia, Valencia, Spain

## Abstract

**Introduction:**

Psychiatry residency programs in Spain offer diverse clinical rotations to ensure comprehensive training. However, certain rotations may require adjustments in length or structure to meet the evolving educational needs of residents. This study assesses the opinions of psychiatry residents regarding which rotations should be extended, shortened, or maintained.

**Objectives:**

To evaluate the perceived need for adjustments in rotation length across various subspecialties of psychiatry, including child and adolescent psychiatry, dual pathology, and psychotherapy, among others.

**Methods:**

Data was collected through a national survey of psychiatry residents in Spain. Respondents were asked to indicate whether specific rotations should be lengthened, shortened, or maintained. Quantitative analysis was performed on responses for seven key rotations: child psychiatry, dual pathology, psychotherapy, research, neuropsychiatry, community psychiatry, and geropsychiatry.

**Results:**

A total of 109 psychiatry residents participated in the survey. The most frequently requested extension was for geropsychiatry, with 57% of respondents advocating for a longer rotation, followed closely by community psychiatry (48%). In contrast, rotations in research (26%) and child psychiatry (24%) were identified as those most needing to be shortened. Most residents supported maintaining the current duration of dual pathology (86%) and neuropsychiatry (83%) rotations.

**Table:** 

Rotation	Shorten (%)	Lengthen (%)	Maintain (%)
Child Psychiatry	24	15	64
Dual Pathology	6	11	86
Psychotherapy	10	17	80
Research	26	22	57
Neuropsychiatry	15	10	83
Community Psychiatry	3	48	55
Geropsychiatry	1	57	48

**Conclusions:**

The results suggest a strong desire among psychiatry residents to extend rotations in geropsychiatry and community psychiatry, while shortening research and child psychiatry. These findings highlight the need for training programs to reevaluate the duration of certain rotations to better align with resident learning needs.

**Disclosure of Interest:**

None Declared

